# Pelvic Tuberculosis Diagnosed during Operative Laparoscopy for Suspected Ovarian Cancer

**DOI:** 10.1155/2018/6452721

**Published:** 2018-04-15

**Authors:** Daniel Martingano, Kayla Cagle-Colon, Jeanine Chiaffarano, Alan Marcus, Diana Contreras

**Affiliations:** ^1^Virginia Polytechnic Institute and State University, Virginia College of Osteopathic Medicine, Blacksburg, VA, USA; ^2^Department of Obstetrics & Gynecology, New York University School of Medicine, New York, NY, USA; ^3^Department of Obstetrics & Gynecology, NYU Langone Hospital-Brooklyn, Brooklyn, NY, USA; ^4^Department of Pathology, New York University School of Medicine, New York, NY, USA; ^5^Division of Gynecologic Oncology, Department of Obstetrics & Gynecology, Atlantic Health System, Morristown, NJ, USA

## Abstract

**Background:**

While the combination of a pelvic mass, very high serum level of CA-125, chest adenopathy, and ascites is concerning for advanced-stage ovarian cancer, the etiology of such a presentation can be due to disseminated pelvic tuberculosis.

**Case:**

A 67-year-old para 2 African-American woman presented with abdominal pain and shortness of breath. Subsequent CT and MR imaging demonstrated chest adenopathy, a pelvic mass, omental caking, and ascites. The patient underwent diagnostic laparoscopy with biopsy revealing noncaseating granulomas and subsequent tissue culture revealed* Mycobacterium tuberculosis.* The patient was diagnosed with disseminated pelvic tuberculosis and multidrug therapy was initiated.

**Conclusion:**

Pelvic tuberculosis can mimic advanced-stage ovarian cancer; thus obtaining a tissue sample may be beneficial to more appropriately direct treatment and planning for neoadjuvant therapies given the ineffectiveness of extensive surgical procedures in treating pelvic tuberculosis commonly employed in the treatment of advanced-stage ovarian cancer.

## 1. Case Report

A 67-year-old para 2 African-American female presented to her primary provider complaining of abdominal pain and distention for 2 weeks, a 10 lb weight loss, and shortness of breath. Past medical history was significant for well-controlled hypertension. Past surgical history included an abdominal myomectomy and two cesarean deliveries. Outpatient abdominal sonogram showed a 1.3 × 1.8 × 2 cm pancreatic head mass with multiple hypoechoic lesions throughout the pancreas, the largest measuring 3.2 cm. Tumor markers were sent and resulted in CEA < 0.5 ng/mL, CA 19-9: 19 U/mL, CA-125: 434 U/mL. Further imaging was ordered including CT scan with contrast which revealed an 8 × 8 × 7 cm pelvic mass, ascites, peritoneal nodularity, and lymphadenopathy. The patient was referred to the emergency department (ED) for drainage of the ascites, additional imaging, and biopsy. In the ED, a CT of the chest showed left cervical, superior mediastinal, left paratracheal, epicardial, and perihepatic lymphadenopathy with a moderate right-sided pleural effusion. A MRI revealed a 12 × 7.5 × 7.8 cm uterus with 2.4 × 2.3 cm right ovarian mass, peritoneal nodules, dense omental carcinomatosis extending into the anterior pelvis, and para-aortic paracaval adenopathy extending from renal hilum to the aortic bifurcation with no visible pelvic adenopathy. This patient was referred to the institution's division of gynecologic oncology where an omental mass biopsy was ultimately performed demonstrating noncaseating granulomas with no tumor identified. The ascitic fluid was negative for malignancy.

**Figure 1:**
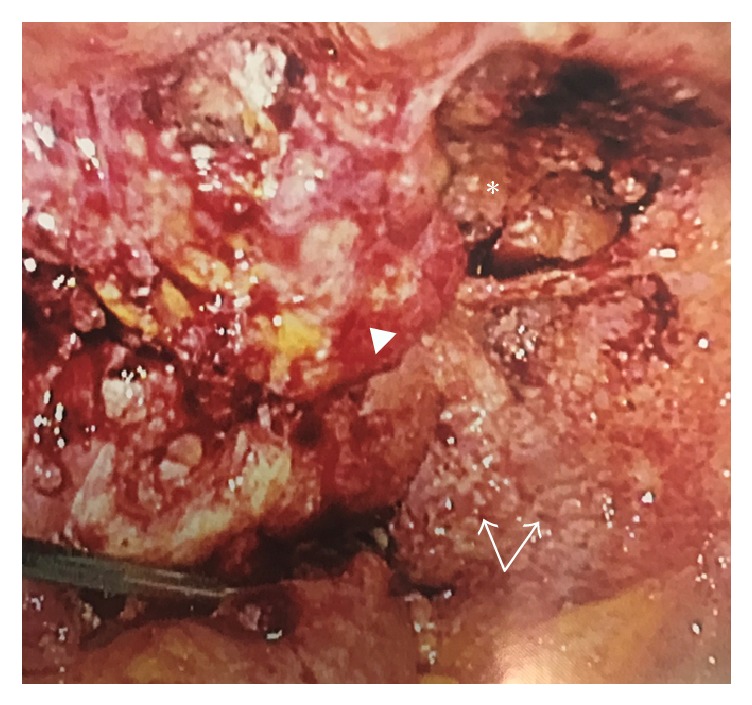
Intraoperative findings of disseminated pelvic tuberculosis: on diagnostic laparoscopy, diffuse disease presumed to represent advanced-stage ovarian cancer which included an obliterated posterior cul-de-sac (*∗*), numerous granulomas (arrows), and distorted pelvic viscera where only uterine body was identified (arrow head.).

**Figure 2:**
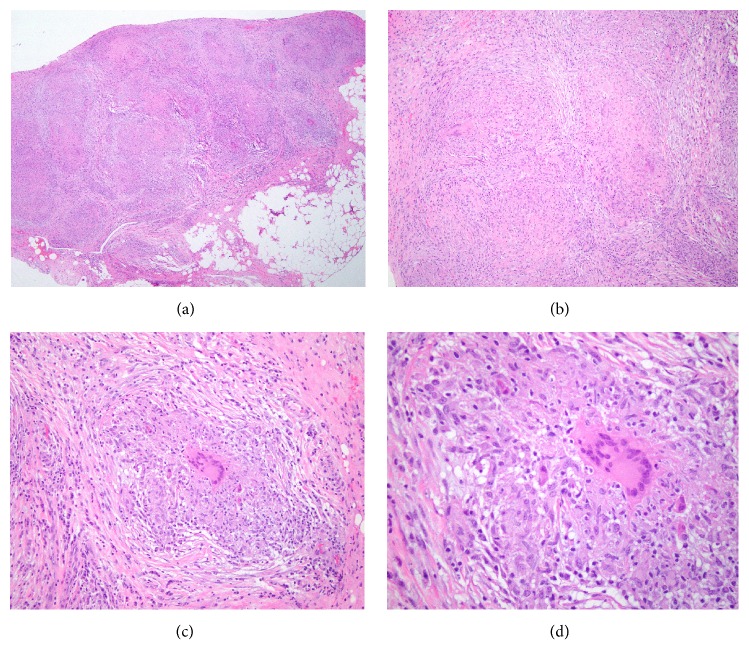
Histopathological analysis. The specimen contains noncaseating granulomata composed of aggregates of epithelioid histiocytes and Langerhans giant cells surrounded by chronic inflammation. The special stain AFB is negative for acid fast organisms and the special stain Gomori Methenamine Silver (GMS) is negative for organisms. (a) Granuloma H&E staining (4x). (b) Granuloma H&E staining (10x). (c) Granuloma H&E staining (20x). (d) Granuloma H&E staining (40x).

The patient was discharged and referred to our division of gynecologic oncology for a second opinion with a suspicion of advanced-stage ovarian cancer. After obtaining a detailed history, decision was made to perform a diagnostic laparoscopy and obtain tissue samples prior to planned surgical treatment. The patient underwent diagnostic laparoscopy and tissue sampling. Diffuse disease representing presumed carcinomatosis was encountered (Figure 1). Subsequent biopsies and frozen section revealed noncaseating granulomas with no evidence of malignancy. Preliminary acid fast bacilli (AFB) stains were negative (Figure 2). Four (4) weeks later, preliminary results from AFB tissue culture were positive for* Mycobacterium tuberculosis* with final confirmation six (6) months after. The patient was subsequently diagnosed with disseminated pelvic tuberculosis (TB) and referred to the division of infectious diseases for combination therapy.

## 2. Discussion

Pelvic tuberculosis is a rare and often difficult disease to diagnose which can present with features that are indistinguishable from ovarian malignancy such as abdominal pain, ascites, and pelvic mass. Due to the rare and deadly nature of ovarian cancer, most women with this presentation are frequently presumed to have advanced-stage ovarian cancer; therefore peritoneal and/or mass tissue acquisition for pathological study is pertinent for confirmation.

Difficulties arise when attempting to preoperatively differentiate between ovarian cancer and TB. The initial evaluation of a patient thought to have pelvic tuberculosis should include a detailed history, including family history of pulmonary tuberculosis, physical examination, Purified Protein Derivative (PPD), and chest X-ray (CXR) if the skin test is positive or if there is a history of Bacille Calmette Guerin (BCG) vaccination. Characteristics of pelvic tuberculosis commonly found on pelvic ultrasound include adnexal mass, tubal disease, ascites, peritoneal thickening, and omental and/or cul-de-sac nodularity [1]. A Mantoux skin test may be nonreactive and mycobacterium difficult to detect in ascitic fluid by smear or culture due to the paucibacillary nature of pelvic disease [2]. Polymerase Chain Reaction (PCR) for* M. tuberculosis* and culture for acid fast bacilli have been used but are not very sensitive and specific in diagnosis [3]. CA-125, a nonspecific epithelial tumor marker, does not discriminate between ovarian malignancy and TB because the elevated levels may be found secondary to the peritoneal involvement in the disease process [3, 4]. However, similar to ovarian cancer treatment, normalization of the CA-125 level has been shown to be associated with the response to anti-TB therapy [3, 4].

The treatment of peritoneal TB and that of ovarian carcinoma differ markedly, necessitating an accurate diagnosis. Diagnostic laparoscopy is currently the principle modality of diagnosing genital tuberculosis because many of the common findings can be seen during the procedure. The most common macroscopic finding is pelvic adhesions, followed by tubal pathology or occlusion, peritoneal, fallopian tube, or ovarian tubercles, perihepatic adhesions, tubo-ovarian mass, ascites, and caseous or granulomatous nodules [5]. Sometimes the diagnosis of pelvic TB can be made instantly when small tubercles (milia) are observed on the peritoneum, but when milia are not present, frozen section is also valuable intraoperatively and can prevent unwarranted, extensive surgery [3].

Due to technical difficulties of techniques to obtain ascitic fluid for preoperative analysis, diagnostic laparoscopy is the gold standard [2]. Laparoscopic biopsies of the questionable lesions are a sufficient and safe method to provide tissue samples for histologic and bacteriologic diagnosis of TB infection with sensitivity > 80%, especially in the presence of ascites [5]. Although laparoscopy appears to be a sufficient and safe method to provide tissue samples for histologic diagnosis of peritoneal or pelvic TB, because diagnosis is not made preoperatively, laparotomy is done often for presumed ovarian malignancy [2]. As ovarian cancer treatment generally consists of radical dissection and preoperative diagnosis of peritoneal TB being difficult, performing laparotomy may be justifiable.

Laparoscopy and hysteroscopy have been used to detect pelvic and abdominopelvic TB. Genital tuberculosis is often found in the fallopian tubes first and most frequently followed by the endometrium, ovaries, cervix, vagina, and vulva [6].

During the reproductive period, caseation is rare in tuberculous endometritis [7]. However, in postmenopausal women, TB granulomas have enough time to develop caseation as there is no sloughing of the endometrium [7]. In the reproductive age, tuberculous granulomas have to regenerate from the basal layer after menstrual shedding of the functionalis layer. The granulomas become well developed and numerous as the menstrual cycle progresses [7]. Endometrial biopsy is therefore recommended just before menstruation or the late secretory phase, as the granulomas get the longest possible time to develop and there is a greater chance of achieving an accurate diagnosis.

Pelvic TB may rarely be directly transmitted sexually with the majority of cases resulting from reactivation of latent TB foci in the peritoneum or from lymphatic or hematogenous spread of primary pulmonic infection; however primary focus in the lungs usually heals completely without any clinic or radiologic evidence making it more difficult to discern between ovarian carcinoma and TB [2].

Knowledge of the clinical and radiologic presentations is paramount for early detection and diagnosis. In the reported case, the patient's vague clinical signs and the combination of radiologic findings of adnexal mass, chest adenopathy, omental caking, and ascites led us to suspect metastatic ovarian carcinoma, but histological findings and culture allowed us to diagnose disseminated pelvic tuberculosis correctly.

The possibility of pelvic TB should be considered in the differential diagnosis of ovarian carcinoma, especially in women who may have been exposed to tuberculosis. Cytologic examination and culture of ascitic fluid obtained by paracentesis may yield a diagnosis; however if ascitic fluid cannot be obtained, or the cytologic examination and culture result are inconclusive, diagnostic laparoscopic biopsy is needed for confirmation. If laparoscopy is impossible, exploratory laparotomy is performed with biopsies taken from intraoperative frozen section examination and samples are sent for culture and PCR testing. If no carcinoma is detected and the diagnosis of peritoneal TB is confirmed, extended surgery is avoided and anti-TB treatment is started. Thus it is imperative that gynecologists and gynecologic oncologists are adept at recognizing pelvic TB to initiate prompt and appropriate treatment.
